# Using Behavior Over Time Graphs to Spur Systems Thinking Among Public Health Practitioners

**DOI:** 10.5888/pcd15.170254

**Published:** 2018-02-01

**Authors:** Larissa Calancie, Seri Anderson, Jane Branscomb, Alexsandra A. Apostolico, Kristen Hassmiller Lich

**Affiliations:** 1 Center for Health Equity Research, Social Medicine Department, School of Medicine, University of North Carolina at Chapel Hill, Chapel Hill, North Carolina; 2 Health Policy and Management Department, Gillings School of Global Public Health, University of North Carolina at Chapel Hill, Chapel Hill, North Carolina; 3 Georgia Health Policy Center, Georgia State University, Atlanta, Georgia; 4 National Maternal and Child Health Workforce Development Center, Maternal and Child Health Department, Gillings School of Global Public Health, University of North Carolina at Chapel Hill, Chapel Hill, North Carolina

## Abstract

Public health practitioners can use Behavior Over Time (BOT) graphs to spur discussion and systems thinking around complex challenges. Multiple large systems, such as health care, the economy, and education, affect chronic disease rates in the United States. System thinking tools can build public health practitioners’ capacity to understand these systems and collaborate within and across sectors to improve population health. BOT graphs show a variable, or variables (*y* axis) over time (*x* axis). Although analyzing trends is not new to public health, drawing BOT graphs, annotating the events and systemic forces that are likely to influence the depicted trends, and then discussing the graphs in a diverse group provides an opportunity for public health practitioners to hear each other’s perspectives and creates a more holistic understanding of the key factors that contribute to a trend. We describe how BOT graphs are used in public health, how they can be used to generate group discussion, and how this process can advance systems-level thinking. Then we describe how BOT graphs were used with groups of maternal and child health (MCH) practitioners and partners (N = 101) during a training session to advance their thinking about MCH challenges. Eighty-six percent of the 84 participants who completed an evaluation agreed or strongly agreed that they would use this BOT graph process to engage stakeholders in their home states and jurisdictions. The BOT graph process we describe can be applied to a variety of public health issues and used by practitioners, stakeholders, and researchers.

## Behavior Over Time Graphs

The chronic disease challenges faced by the public health workforce in the United States and US jurisdictions are embedded in complex systems. Systems are composed of heterogeneous elements and their interactions (1). The dynamics of cause and effect in complex systems are difficult to identify because of the many interactions between contributing factors and across socioecological levels over time (2). Complexity makes it difficult to fully understand a system, much less mentally simulate the likely trajectory of outcomes under various intervention scenarios (3). Systems thinking, or “the ability to see the world as a complex system” where “everything is connected” (4), offers an approach for addressing complexity in systems. We describe a systems thinking technique for facilitating discussion, shared understanding, and consensus among groups of diverse collaborators working to reduce the high burden of chronic disease in the United States.

Systems thinkers often create Behavior Over Time (BOT) graphs as an initial step to understand a complex system. BOT graphs are well-established components of group model building in system dynamics but are useful in many other contexts (5,6). BOT graphs are typically constructed early in the planning phases of a research study, project, or program to formulate a research question, understand the problem of interest, draw out participants’ mental models, and generate and compare hypotheses about key determinants of the problem and strategies for action (7,8). BOT graphs are plots of one or more variables (*y* axis) over time (*x* axis). In a BOT graph group activity, participants are asked to graph a variable or variables that they think are important to the challenge being studied or that capture the behavior of the system over time and to describe their graphs to the group (6). Creating BOT graphs, also known as a reference mode activity, has been in widespread use in the group-modeling building community for years (5–13).

The purpose of this article is to demonstrate that BOT graphs are tools that public health practitioners can use to engage diverse groups to analyze and discuss complex challenges. We describe how BOT graphs are used in public health; how they can generate discussion among groups, including stakeholders; how they were used with a group of maternal and child health (MCH) public health practitioners and partners in the United States; and how BOT graphs can advance systems thinking.

## Behavior Over Time Graphs in Public Health

BOT graphs, also called time series or trend graphs, are common in public health. Epidemiologists present longitudinal data in this format, and public health problems such as rates of smoking (14), childhood obesity (15) and teenage pregnancy (16) are often communicated using trends over time. Decision-makers, such as health department leaders and policy-makers, receive BOT graphs in reports and presentations to inform their decision-making processes. For example, a health department might set a goal to decrease the slope of increasing obesity trends among specific populations in a 5-year period.

In group model building, BOT graphs are used to understand a problem from multiple perspectives. For example, an epidemiologist may draw smoking rates as rising and falling over the past 50 years, but a young person might perceive smoking rates among his or her friends to have been rising steadily since the introduction of e-cigarettes. Considering multiple perspectives can provide a more holistic view of a problem. Observed data should be used to create BOT graphs when they are available. However, participants are not limited to drawing BOT graphs of variables for which observational data can be found. The group activity of creating a BOT graph is a way of identifying key variables and discussing what drives changes in those variables over time. Because of the complexity of systems, some variables are difficult to measure or have not been previously identified as critical data to collect (6). When data are limited or unavailable, BOT graphs containing perceived factors and trends could provide insights and inform future data collection priorities (7). Conflicts between observed data and perceived trends should be discussed to understand why the discord exists. Doing so is a learning opportunity for participants (7).

## Using Behavior Over Time Graphs to Generate Discussion

BOT graphs can be used to engage diverse stakeholders in discussions about public health challenges. Stakeholders can be people experiencing a challenge first hand, such as a group of people with type 2 diabetes aiming to improve their blood glucose levels through self-care, or groups who work with or support that population, such as doctors, dietitians, diabetes educators, researchers, or family members. BOT graph discussions can occur with a homogenous group (eg, people with diabetes) or a heterogeneous group (eg, people with diabetes, diabetes educators, doctors, family members). Topics covered and views exchanged during the discussion vary depending on who participates and how broadly the discussion is framed.

The 5 steps for using BOT graphs build shared understanding of the dynamics of a selected challenge. First, the group defines the focal challenge. Second, each individual selects an indicator reflecting the challenge. Third, each individual sketches and annotates the trend. Fourth, individuals extend their trend lines into the future, predicting what could happen under various scenarios. Fifth, the group discusses why participants drew the shapes they did and explains which system forces most impact trends. These steps are adapted from the Graphs Over Time script described in the group model building methods clearing house, Scriptapedia (6,17). Planners of BOT graphing sessions typically spend at least 15 minutes describing the process and 45 minutes on the activity (6).

**Step 1**. In the first step, the group confirms that there is a shared understanding of the public health challenge they are meeting to address. Participants in BOT discussions should determine the scope of the challenge and then define that challenge as specifically as possible within the agreed-upon scope. A broad scope might be for a state to improve access to children’s health care services whereas a narrow scope might be to increase use of a Medicaid managed care navigation program in a specific region. Defining a target population and region may help the group determine the scope of the challenge. Complex challenges work well for BOT graph discussions because they are influenced by many interrelated factors and can influence varied outcomes (5).

**Step 2**. In the second step, the group selects a variable or variables of interest to plot on the *y* axis and a relevant timeframe to plot on the *x* axis. Selecting these variables, either outcomes of interest or other factors that contribute to them, begins to indicate how stakeholders with different experiences and knowledge think about and understand the challenge. For example, in a group of stakeholders focusing on children’s access to health care services, one participant might graph the percentage of children who have health insurance, while another participant might graph the percentage of parents who are satisfied with their child’s health care services. Participants would be encouraged to draw the variables that most meaningfully describe the problem or are most important in determining change over time and then share their selections with the group. The dynamics of multiple variables (eg, an outcome and the factors driving that outcome) can be drawn on the same graph, or they can be drawn separately and later superimposed onto a single graph; this approach helps participants hypothesize mechanistic explanations for their BOT graph. This step can also help groups develop shared measures, a principal component of a collective impact approach (18).

**Step 3**. After each participant selects a variable and relevant timeframe, the third step is for each participant to draw a BOT graph. Data, such as percentage of people with diabetes with controlled hemoglobin A1c, percentage of the population in India with access to clean drinking water, or breastfeeding rates by race/ethnicity group in the United States, should be used to plot the BOT graph trend lines if data are available. However, in the absence of data, given the intention of drawing out participants’ perceptions of what factors are driving change, participants are encouraged to depict their perceptions of how their variables of interest have changed over time. Some variables, such as equity, are concepts that are not easily measured. In such cases, participants can draw trend lines of concepts as they understand them, or participants can select indicators to operationalize their understanding of a concept that is difficult to measure. Selecting, drawing, and explaining trends of indicators related to a concept can help participants develop a more nuanced view of that concept and how it relates to outcomes of interest. Moreover, participants might learn about measures they did not know existed as they share their BOT graphs with the group, or they may brainstorm measures they wished existed. Participants are encouraged to draw general trends (increasing, decreasing, oscillation of, or no change) over time for variables, even if they do not have data available. The goal is for the BOT graphs to serve as a starting point for discussion, inspiring hypotheses about what determines patterns over time.

**Figure 1:**
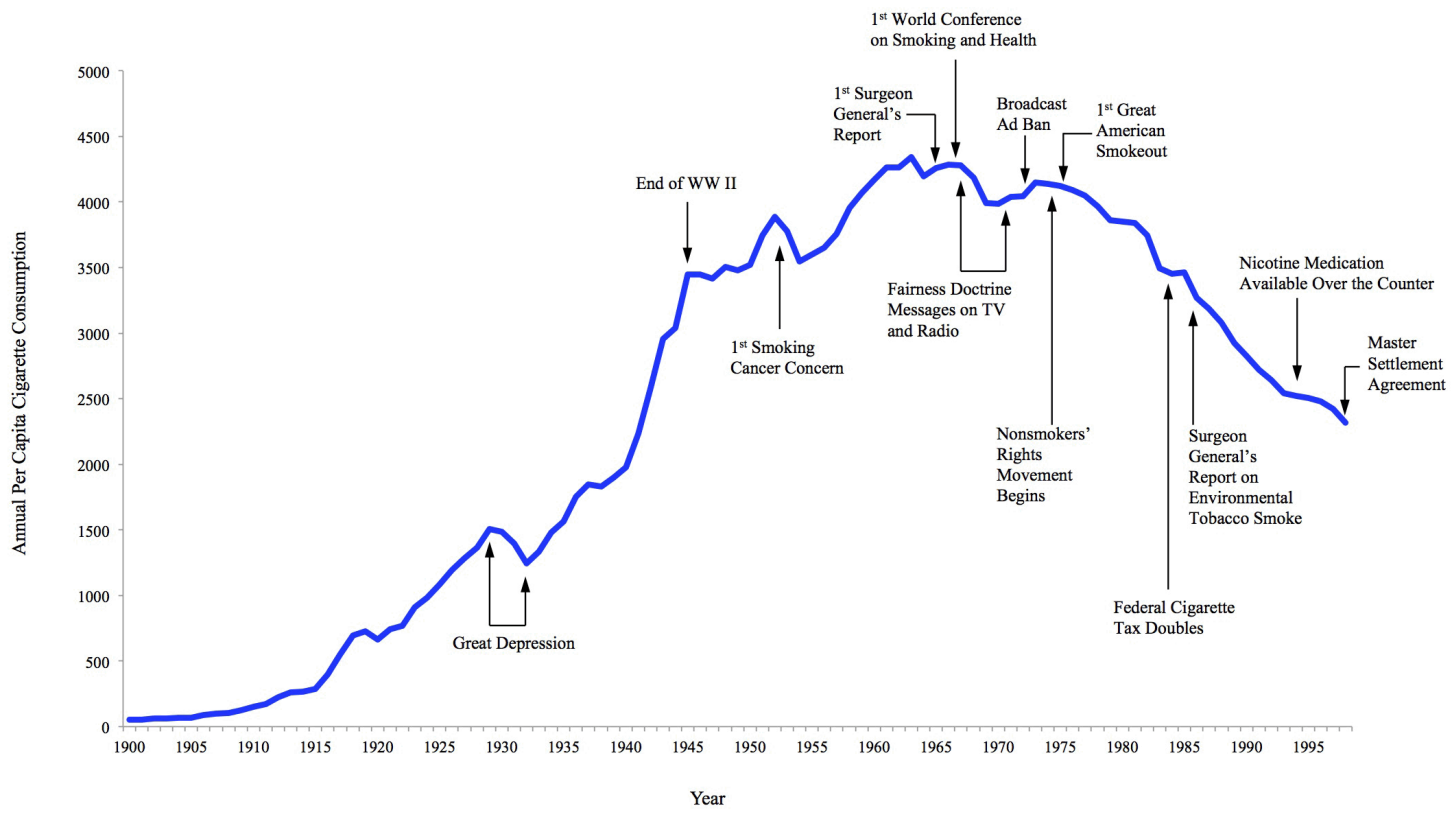
Annotated Behavior Over Time graph that shows annual per capita number of cigarettes consumed and major smoking and health events in the United States from 1900 to 1998 (14).

As participants graph their variable or variables over time, they should annotate their graph with the factors that they believe affect the trend line. Annotations could refer to inflection points in the graph, perhaps aligning with fast-acting interventions such as a quick drop in smoking prevalence following a ban on smoking indoors, or to sections with a common slope, such as a gradual downward trend in smoking prevalence as public awareness that smoking has negative health consequences grows (14). Participants should share their annotated BOT graphs with the group, explaining selected variables, choice of timeframe, general trends over time, and the key factors they hypothesize are most responsible for driving those trends over time. Systems thinking tools such as BOT graphs encourage users to consider 4 levels of thinking: events, patterns, systemic structures, and mental models (1,19). For example, Figure 1 presents an annotated BOT graph of annual per capita cigarette consumption among adults in the United States over time (1900–1998) and events likely to contribute to trend fluctuations over time (14). A discussion about those events and trends could indicate the systems that shape health policy making in the United States and the mental models, or beliefs, values, and assumptions, informing policy-making systems (19).

**Step 4**. The fourth step is to ask participants to project what trends they might see in the future under different conditions. The conditions could be business as usual, a response to an intervention or policy, or a response to some other event they believe to be important, such as an economic recession. Participants should draw and annotate their trend projections and share their scenarios with the group.

**Step 5**. The fifth step is to ask participants to share their graphs with the group. Facilitators should encourage participants to discuss the events, patterns, systemic structures, and mental models that are likely to drive the trends participants identified. Although the resulting BOT graphs may be useful to illustrate trends over time, the discussion and learning that occur during a group BOT graphing session may be more valuable.

## An Example of Behavior Over Time Graph Discussions In Action

We used BOT graphs to generate discussion about complex MCH challenges among groups of 101 participants and their partners from across the United States at the National Maternal and Child Health Workforce Development Center Skills Institute in August 2016. The National Maternal and Child Health Workforce Development Center, funded by the Maternal and Child Health Bureau, provides Title V state and jurisdiction leaders, staff members, and partners from other sectors with opportunities to develop skills in evidence-based decision making, systems integration, and change management to improve the health of women and children (20). Most participants were Title V leaders and staff members (62%) (Table 1). Partners included MCH professionals from other state health departments (14%), parent and family representatives (14%), health providers and professionals (5%), and representatives from hospital and health care systems (3%), and from Medicaid (2%). Thirty-six teams participated in the Skills Institute. Each state or jurisdictional team arrived with a particular MCH challenge, drawn from MCH national performance measures, national performance outcomes, or state-specific outcomes (21).

**Table 1:** Participant (N = 101) Affiliations, National Maternal and Child Health Workforce Development Center’s Skills Institute Session on Behavior Over Time Graphs, 2016

Census Region	No. (%)	Agency Affiliation	No. (%)
Northeast	7 (7)	Title V	5 (71)
		State health department	2 (29)
Midwest	18 (18)	Title V	10 (55)
		Family advocacy	2 (11)
		Health provider	2 (11)
		State health department	2 (11)
		Hospital/health systems	1 (6)
		Other	1 (6)
South	43 (43)	Title V	24 (56)
		Family advocacy	7 (16)
		State health department	7 (16)
		Medicaid	2 (5)
		Other	2(5)
		Health provider	1 (2)
West	19 (19)	Title V	11 (58)
		Family advocacy	5 (26)
		State health department	2 (11)
		Other	1 (5)
US territories	14 (14)	Title V	10 (72)
		Health provider	2 (14)
		State health department	1 (7)
		Other	1 (7)

The goal of the 2-hour BOT graph session was to introduce the tool and then allow teams to use BOT graphs to discuss trends related to their MCH challenge area. First we provided a brief overview of what BOT graphs are and how they can be used (10 minutes). The presenter used a large paper to sketch a simple version of the BOT graph (Figure 1) to explain how to create and annotate the graphs. The group was asked to draw BOT graphs, related to one of 2 example prompts (percentage of children living with a smoker, or percentage of pregnant women who smoke while pregnant) (15 minutes). We provided dry erase markers, tissues, and erasable plastic sheets resembling white boards. Presenters circulated around the tables, encouraging participants to diagram trends, even if they did not have data or dates on hand. After several minutes, participants shared their BOT graphs with participants from other state teams (10 minutes). Together, the group identified various universal, state, and national factors that influenced these variables over time.

**Figure 2:**
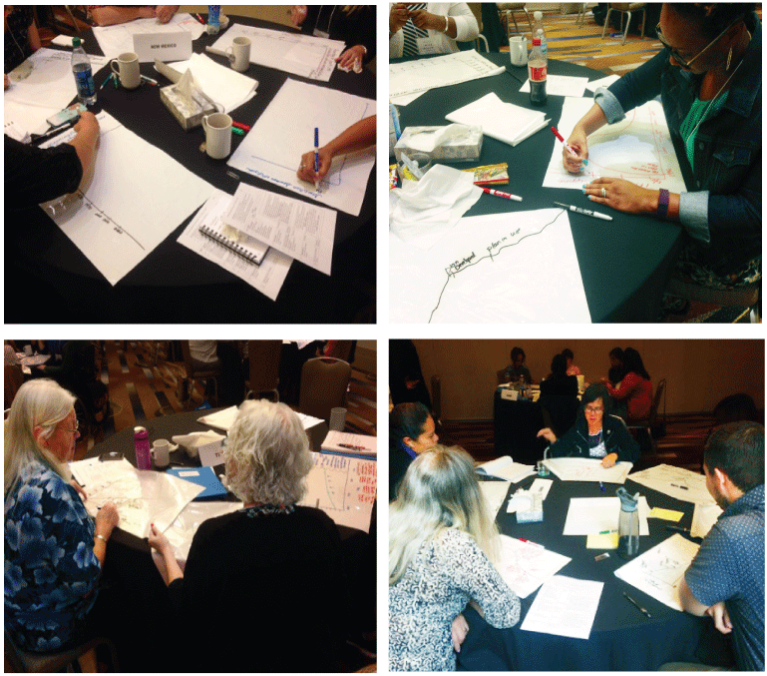
Photographs of maternal and child health practitioners and partners working on behavior over time graphs at the National Maternal and Child Health Workforce Development Center’s 2016 Skills Institute.

Next, the state teams discussed their focal challenge and what variables were related to that challenge (10 minutes). Team members independently drew BOT graphs (Figure 2). Next, we asked participants to include trends showing how the variables could change in the future in response to an intervention, policy change, or other scenario (15 minutes). At some tables all members drew the same variable, while at other tables, members of the same group drew different variables over time. For example, in one group all participants graphed participation in a preschool health promotion program over time. At another table, group members each thought about variables that reflected racism in their state, a challenge they agreed affected MCH outcomes in their target population, and annotated BOT graphs showing trends in racism over time. That particular group struggled with indicators that capture racism, a complex social construct, which led to interesting discussions within their team. While the team did not have time to definitively select indicators during the BOT session at the Skills Institute, they started the process of identifying root causes related to their MCH challenge and seeking indicators that reflect those key factors. An alternative approach to asking participants to select variables would have been to provide data about a variable over time and ask the group members to graph 1 or 2 factors that were contributing to the observed data trends, annotating key determinants of those factors (22). Insight can grow when the group integrates their individual graphs to develop a consensus graph that contains multiple variable trends graphed over the same period (10 minutes to discuss). This process requires participants to critique and integrate each other’s understanding of determinants of the BOT graph. The BOT graph exercise encouraged individual and collective systems thinking to advance the teams’ understanding of a complex MCH challenge.

To conclude the BOT graph session, we asked participants about their reactions to the process of using BOT graphs as a discussion tool (10 minutes). They provided verbal feedback to the group at the end of the session, and 84 participants provided anonymous written feedback on an evaluation survey. Participants described BOT graphs as practical, innovative, and easy to grasp (Table 2). The themes reflect how this process can advance systems thinking. Seventy-two evaluation survey participants (86%) said they agreed or strongly agreed that they would use this process to engage stakeholders back in their home states and jurisdictions.

**Table 2:** Themes and Example Quotes From Practitioners and Partners, National Maternal and Child Health Workforce Development Center’s Skills Institute Session on Behavior Over Time Graphs, 2016

Theme	Example Quote
BOT graphs helped Skills Institute participants expand their thinking related to a complex challenge, helping them see the bigger picture.	“It’s a unique way to brainstorm about the issue. It makes you think a little bit differently, especially when you share them as a group.”
	“It helps you do anticipatory planning. If you think something’s going to happen, how are you going to design the system now so the outcome is impacted in a certain way [in the future].”
	“[BOT graphs] really allowed me to see how environmental and social factors affect health.”
BOT graphs allowed users to better understand each other’s mental models.	“I like the idea of having people drawing their perspective and clarifying mental [models].”
	“This process omitted the ‘assuming’ factor when you are sitting at the table talking to other partners or co-workers. . . . Looking at this picture, it helps me see why they are saying what they are saying, because it’s very different from how I look at it.”
	“Assuming can be dangerous. It can get you stuck so you can’t get to your end goal with your partners.”
Projecting future trends allowed participants to be optimistic about their role in the complex challenge.	“[When I draw future trends] I can *see* how my plans made a difference.”
BOT graphs can function as a communication tool with community members.	“You get their [community members’] knowledge base and their perspective . . . it’s more of a participatory thing based on what they know and their values. It’s not so academic [when you ask them to estimate and describe trends rather than use real data].”
	“For me, it was helpful to draw a picture . . . and letting that be your story, sort of like qualitative research.”

Abbreviation: BOT, behavior over time.

## Advancing Systems Thinking By Using Behavior Over Time Graphs

When public health practitioners use BOT graphs as a discussion tool, they develop a deeper understanding of a complex problem by hearing others’ perspectives (9). This process can bring stakeholders together to diagnose a shared problem (23). Stakeholders voice elements or interactions within a system, leading to a more holistic “big picture” view of a challenge (5,22). Systems thinking encourages holistic thinking, rather than shrinking a challenge into a small set of variables, as is often the case with many scientific methods (5,24). Systems thinking also encourages transcending events to determine trends, systemic structures, and mental models that produce events (1). In addition to holistic, deep thinking, systems methods such as BOT graphs may illuminate structural elements of a complex system, including relationships between factors, feedback loops, and delays between cause and effect (5,25). Using systems science tools such as BOT graphs, system dynamics models, and network analysis can help public health practitioners understand, describe, and intervene within complex systems to address public health challenges (2). A major goal of applying systems thinking in public health is to develop comprehensive, practical solutions to complex challenges that influence population health outcomes (26–28). Integrating many perspectives into a big-picture understanding of a challenge allows public health practitioners to think creatively about how to address a challenge and what the intended or unintended consequences of their actions could be (28).

Public health practitioners can use BOT graphs to develop a big-picture view of a challenge, select outcomes that are important indictors to different groups of stakeholders, examine how outcomes change over time, and identify factors that may contribute to those changes (5). BOT graph activities can also help groups understand and communicate about how various evidence-based strategies could influence public health trends. Doing so can aid groups in selecting effective interventions and strategies. Group BOT graph activities provide opportunities to better understand one another’s mental models, or perceptions about the factors and the relationships between those factors related to a certain topic (7,22,23). This process advances systems thinking. BOT graphs and other systems science tools can help public health practitioners address complex, multifaceted and multilevel challenges facing the population.
